# Uric Acid in Its Relation to Disease, with Special Reference to the Diseases of the Oral Cavity

**Published:** 1901-08

**Authors:** J. C. Bierwirth

**Affiliations:** Brooklyn, N. Y.


### URIC ACID IN ITS RELATION TO DISEASE, WITH SPECIAL REFERENCE TO THE DISEASES OF THE ORAL CAVITY.[1]

BY J. C. BIERWIRTH, M.D., BROOKLYN, N. Y.

In selecting the above caption for this paper, the writer wishes to state, at the outset, that this is done for brevity chiefly, as it does not express the scope intended; for to treat of uric acid and all the diseases it is supposed to cause in the short time at our disposal is an impossibility, and we will therefore have to content ourselves with a brief and sketchy outline only, so as to introduce the subject for a general discussion.

For several years the writer has been impressed with the widely prevalent functional disturbances and organic diseases traceable to a faulty elimination of the products of tissue waste. Many works have been written on the two most prevalent diseases belonging to this group,—namely, rheumatism and gout,—and many theories have been advanced as to their causes, but in most text-books very little can be found which would throw any light upon the more obscure functional disturbances of the various organs of the body resulting from autointoxication. With the ever-increasing knowledge of the chemistry of the body, we are enabled now to recognize many disease processes as being due to autoinfection, or, to speak more correctly, being endogenetic,—*i.e.*, produced within the body, and not due to some obscure outside cause. Viewed from this stand-point many diseases have become clear to us, and we are enabled to treat them intelligently. The writer has given much thought and study for the past eight or ten years to the diseases under consideration, he is convinced of the correctness of the above expressed opinion, and it has been a source of much gratification to prove clinically the results of the laboratory experiments. The more we think of the endogenetic causes, as factors in the production of disease, the better will we be able to diagnosticate and treat successfully hitherto but little understood functional disturbances and organic diseases.

In order to better understand the subject, it will be necessary to review briefly the chemistry of the various products of tissue

---

[1] Read before The New York Institute of Stomatology, April 2, 1901.

waste from their probable source to the final compounds as they appear in the urine. We cannot entirely subscribe to this statement of Purdy: "The variations in nutrition and waste are accurately recorded in the urine, hour by hour and by an intelligent interpretation of modern methods of urine analysis these physiological tides may now be read as accurately as we can number the pulsations of the heart." It is no doubt true that the variations in nutrition and waste are accurately recorded in the urine, but physiological chemistry has thus far not sufficiently advanced to enable us to interpret with accuracy either the physiological or pathological tides of nutrition. So long as we are unable to follow with accuracy the various chemical processes, from the food as it enters the stomach, through its complex steps of digestion and assimilation, and final deposit in the tissues, to the ultimate use these tissues make of it, and then on to the final products of waste as found in the urine, so long will our conclusions based upon an analysis of the urine be shrouded in doubt, our diagnosis and prognosis be open to question, and our treatment be largely empirical. But, although our knowledge is not as mathematically correct as Purdy would lead us to believe, we can do a great deal with what we have, and we may hope that the near future may bring such a utopian state as the above quotation indicates.

Nearly the entire product of tissue waste of the body is excreted by the kidneys in the form of urea, uric acid, xanthine, hypoxanthine, creatin, creatinin, and the compounds of oxalic acid. Of these, urea represents about ninety per cent, the remaining ten per cent. being divided among the other compounds. They all belong to a group classed as nitrogenous compounds, and are all corelated chemically.

*Urea,* of the formula $CH_4N_2O$, is the most important, by virtue of its greater quantity. It is derived chiefly from the general tissue waste, and to some extent from the unassimilated nitrogenous food. The liver is now considered the chief laboratory for the formation of urea, with perhaps some aid from the spleen. Clinically this is proved by a considerable increase above normal in diabetes mellitus, in which disease the liver is stimulated to increased action, while in all diseases of the liver, resulting in diminished action of that organ, urea is markedly decreased. The daily amount excreted is variously stated to be from three hundred to six hundred grains in a healthy adult; occupation, sex, and age produce, however, wide variations within the limits of health.

*Uric acid,* having the chemical formula of $C_5H_4N_4O_3$, is constantly present in the urine, but only in very small amounts. Most works on urinalysis state the daily amount to be from eight to twelve grains, but more recent investigations, with the aid of perfected methods, give the amount as from twenty to thirty grains. It is chiefly excreted in combination with several of the elements, notably sodium, as neutral and acid urates. It is to the presence of the acid sodium urate that the urine owes its acidity, and not to uric acid, as is not infrequently believed. The fact that crystals of uric acid are deposited in urines upon standing is no proof that it is present in excess. These crystals are the result of the splitting up of the urates through the so-called acid fermentation by the aid of the mucus present. Uric acid is but slightly soluble in cold water, the proportion being 1 to 15,000, hence it appears in the urine chiefly in the form of the soluble neutral urates, by combining with the various salts in the blood. The acid sodium urate is only sparingly soluble, and is therefore present in only small amounts. Uric acid, like urea, is chiefly formed in the liver, and like it is the result of tissue waste. In the normal state the blood only contains the faintest trace, but in gout and rheumatism the proportion is said to become quite appreciable. It is assumed that uric acid, after its formation in the liver, combines at once with sodium, potassium, and ammonium, and forms neutral salts with these bases, which then are carried by the blood-current to the kidneys for excretion. The acid urates, also called biurates, are found in gouty and rheumatic deposits about the joints. By a further grouping of molecules we get another class of compounds, called quadrurates. These are much more soluble than the biurates, and no doubt exist in the blood. By some of the more recent investigators it is believed that the normal existence of uric acid in the blood is in the form of quadrurates. Roberts considers that all pathological phenomena usually ascribed to uric acid are due to secondary changes in these urates. He has carefully investigated the solubility of the acid urates, notably the sodium urate, in cold water impregnated with various salts, and has tabulated his results in an interesting manner. This solubility of the acid urates has an important bearing upon the treatment of the various disorders due to retained excrementitious materials. Ralph, in his "Practical Treatise on Diseases of the Kidneys," has tabulated the solubility of the urates in cold water as follows: Potassium, acid 1 to 800, neutral 1 to 44; sodium, acid 1 to 1200, neutral 1 to 77; lithium, acid 1 to 60; calcium, acid 1 to 600, neutral 1 to 1500; ammonium, acid 1 to 1600. It is interesting to note from these figures the great solubility of the acid lithium urate, it being 1 to 60, as compared to the feeble solubility of the potassium and sodium urates of 1 to 800 and 1 to 1200 respectively. This greater solubility of the lithium urate has an important bearing upon the treatment of gout and rheumatism, and the therapeutic results obtained from the various lithium salts have amply proved the laboratory results. It will be of interest here to note the probable chemistry of gout and rheumatism. As already stated, uric acid after its formation in the liver, probably at once combines with the alkaline carbonates in the blood, forming the soluble quadrurates, which pass into the blood-current and are excreted by the kidneys. In perfect health this process is carried on with sufficient promptness to prevent detention and accumulation in the blood. But in persons prone to attacks of gout or rheumatism, either from an excessive production of uric acid or a diminished action of the kidneys, or both, the quadrurates accumulate in the blood. This detention results in their transformation into biurates or acid urates, which, being feebly soluble, are soon deposited in various parts of the body and produce the symptoms of gout and rheumatism. It would of course be an assumption to claim that this is all there is to the chemistry of the diseases under consideration; far from it. There are many links yet missing to complete the chain of proof, but in the main the above outline seems to point the way which may lead to the discovery of the true cause. It will now be proper to ask, what causes this faulty chemistry which leads up to a state of disease? Among the investigators of this class of diseases many divers opinions have been expressed. Some claim that they can be explained entirely from the chemical stand-point; others disregard this, and claim that they are due to bacterial infection, and the number in this class seems to be growing every year. To the writer it seems that there can be no doubt that the cause lies somewhere in the gastrointestinal tract; whether a hyperacidity of the stomach of long standing due to nervous causes or chronic indigestion, or both, or whether a bacterial infection in the intestines, causing the production of putrefactive gases, which, being absorbed by the blood-current, disturb the normal chemistry, or whether other yet unknown bacteria in the intestines act as specific causes, are questions which the future will solve, no doubt. When we try to explain a case of acute inflammatory rheumatism on the purely chemical theory, it would seem that something is lacking, and that some more specific cause must exist, which some of the adherents to the bacterial theory seem to have found in the bacillus coli communis.

*Creatin* and *creatinin* are two interchangeable bodies, and are represented in the urine chiefly by the latter, which is an anhydride of the former. Creatinin is a constant constituent of normal urine, and varies in amount from 0.5 grain to five grains, according to the amount of proteid substances ingested. It is of interest here to note that one investigator has recently claimed to have produced an arteriosclerosis in animals by feeding to them large quantities of creatinin, and, arguing from this, is of the opinion that this body is largely responsible for a like condition of the arteries in man.

*Xanthine and hypoxanthine* are of interest, in the present consideration of the subject, only from the fact that they are in reality uric acid less highly organized, having one and two atoms of oxygen less respectively. The formula of xanthine is $C_5H_4N_4O_2$, and that of hypoxanthine is $C_5H_4N_4O$. But little is known of their physiological or pathological effects in the human economy. Some recent observers are of the opinion that, owing to a faulty action of the liver, uric acid passes into the circulation in the less organized form of these two bodies, which, when in excess, act as poisonous.

*Oxalic acid* and its salts have an important bearing on many functional disturbances, and their probable chemistry is of special importance in this connection. Oxalic acid is very largely introduced into the system through the vegetable foods, especially rhubarb, tomatoes, cabbage, spinach, asparagus, etc. In the laboratory of the liver it is readily split up into $CO_2$ and $H_2$, its chemical formula being $C_2H_2O_4$, which would make $2CO_2 + H_2$. Were this not so, the majority of vegetables would act as poisonous, for oxalic acid is one of the most corrosive poisons known. When the digestive functions are carried on normally, the greater portion is split up as shown, and what remains forms a salt with the calcium, and is excreted by the kidneys as calcium oxalate, but when disturbances of digestion occur and the latter is found in the urine in excess, then symptoms of poisoning may be manifest. Oxalate of lime is kept in solution in the urine by the acid sodium phosphate, when present in normal amount, but when present in excess is crystallized out, owing to its insolubility in water; it is freely soluble, however, in hydrochloric and nitric acid. The mere presence of an excess of crystals of oxalate of lime in the urine is of necessity no evidence of a pathological condition, for they are readily formed, during the process of fermentation, by a splitting up of uric acid and urates. It is also perfectly consistent with health when we find them in freshly voided urine of persons who have eaten largely of vegetables rich in oxalic acid. But when we have a specimen of urine which, being protected from fermentation, deposits large quantities of urates, uric acid *and* oxalate of lime, we are justified in considering it pathological, and in applying this knowledge in the treatment of our patients.

If we now turn to the clinical picture, which presents itself to us as the result of this faulty chemistry and the endogenetic diseases caused thereby, we find that it includes almost every organ and system in the body, and shows so varied an aspect in its symptomatology that it seems impossible to give even an outline tracing of it here. The terms uric acid diathesis, lithæmia, oxaluria, gouty and rheumatic diathesis, etc., have all been employed by various writers to designate the ever varying group of symptoms under consideration. These disturbances of health are rarely found in children or young adults, except in families with marked hereditary tendencies, and are mostly met with in persons past thirty-five years of age. Males are more subject to them than females, except, again, where there is a marked heredity. The nervous system seems to suffer most. Severe and frequent attacks of headache, often accompanied by vertigo and vomiting, neuralgias of the various nerves, facial, dental, supraorbital, occipital, and sciatic, are all of common occurrence; sleeplessness at night and drowsiness in the day-time, great irritability of temper, a sense of general prostration with mental hebetude and attacks of melancholia not less frequently. Many complain of severe aches and pains all over the body, described by some as bone-ache, some of areas of hyperæsthesia, especially over the lumbar region, buttocks, and posterior aspects of the thighs, together with attacks of lumbago and a sense of weight over the kidneys. The skin suffers in many patients. In some it is only a general roughness and dryness, in others intense itching is added; again we find patches of dry and moist eczema with or without itching, fissures between the toes with intense itching, dry and brittle toe-nails, and a corrugated and striated condition of the finger-nails. Severe attacks of urticariá, often of a most obstinate character, are not infrequent. The scalp is often dry with a tendency to dandruff and a malnutrition of the hair. The digestive system suffers in the majority of patients; and if we pause here a moment and consider the symptoms in the oral cavity, we will find that here we often get the earliest manifestations. The mucous membranes throughout the body show the earliest evidence of a vitiated blood-current, and that of the mouth is the one where we expect to find the forerunner of many systemic diseases. In the present instance we have an engorgement of the capillaries, especially the veins which give to the gums a bluish tint; where this condition is more marked the gums bleed easily, and if it goes on unchecked a loosening of the gums from the teeth takes place. Pus germs now find their way under these loose gums, and, being nourished by the exuding blood and serum, grow and multiply, and we have the disease pyorrhœa alveolaris. In those patients where the pus germs find no lodgement, atrophy and recession of the gums take place, a condition so frequently seen in persons past middle life, and always due to an improper nutrition. Erosions and decay of the teeth are due to the general malnutrition. How far the saliva and its changed chemical composition influence caries of the teeth the writer is not prepared to state, but he is of the opinion that it must have a decided effect. The actual cause of caries has seemed to him to lie in the saliva, supplemented by a lowered resistance in the teeth to decay, due to their impaired nutrition. The sulphocyanide of potassium in the saliva is a substance which probably varies greatly in amount in different states of health, and is a body, chemically considered, which might easily form corrosive compounds. But little is known thus far about this matter. Neuralgias of the dental nerves and a simple aching of the teeth, pains in the joints of the jaw and the muscles of mastication, fissures in the corners of the mouth, and erosions of lips, gums, and palate may all be caused by the diseases under consideration. Considering further the digestive system, we find in the majority of patients either a constantly coated tongue, or it is red and glazed; the appetite is often poor and capricious, and in many there is an absolute distaste for food. The stomach digestion is slow, with tendencies to fermentation and acid eructations. The bowels are constipated in most patients. In many there is a decided anæmia, especially in those where these disturbances have existed for a long time, and this anæmia yields but sluggishly to treatment. The complexion is often muddy and of a grayish appearance, with muddy conjunctivæ. The eyes may be the seat of repeated attacks of conjunctivitis, and of iritis. The vision is not infrequently disturbed, as shown by inability to read long by artificial light. The nose and throat show a ready tendency to congestion and inflammation of the mucous membrane,—so-called rheumatic angina is a good illustration.

Patients go on to suffer from many of the above-enumerated symptoms for a long time, being usually better in the summer than in the winter on account of the greater activity of the skin and more rapid elimination, but are rarely ever entirely well. They not infrequently consult a physician for the most prominent symptom, either indigestion, headache, or constipation, and are temporarily relieved. They often consult a specialist for their headaches and neuralgias, or for eczema or inflamed eyes, to be helped for the time being, only to have recurrences after a short time of their old troubles. Some consult us for a pain over the kidneys, and, seeing visions of Bright's with the commonly accepted fatal ending in the near future, have become frightened and seek advice. Upon questioning, they usually unfold a very true picture of a faulty chemistry of long standing. Perhaps the largest number of cases seek advice for persistent dyspepsia and constipation, and are relieved by appropriate treatment promptly enough, but they are not cured, and gradually relapse into their former condition, if the true cause is not recognized and eliminated. As stated above, all the enumerated functional disturbances find their origin in an improper food-supply plus an imperfect oxidation. More fuel is put into the machine than it can burn, and soot and smoke soon clog up the flues. It is both the quality and the quantity of food that does the mischief, and its imperfect oxidation is the result of insufficient exercise in the open air. Now, these two main causes would seem to be easily corrected, for it should not be difficult to prescribe a proper dietary and sufficient exercise, but, as a matter of experience, we find that this is the most difficult part of the problem, especially if we bear in mind the weakening effects of long heredity on the organs involved, and also heredity as to habits, which is perhaps an even greater enemy. I firmly believe that heredity plays a most important factor in the conditions under consideration, for we often find many of them represented in the different generations of one family. The quality of food should be considered first of all. Looking at the chemistry of the waste product as found in the urine, we find that they all belong to the nitrogenized compounds, and that they are not only the result of tissue waste, but also represent the unassimilated food-supply. The nitrogenized foods are chiefly meats, fats, eggs, and a few vegetables. Chemical analyses of the fæces of persons of widely differing habits and vocations have shown that in all those taking but little exercise from one-fourth to one-half of the food taken passes off in the fæces unassimilated, while in those performing arduous physical labor the fæces consist only of undigestible refuse. We can readily understand, therefore, that over-assimilation must take place from the intestine filled to overflowing with food. All this food assimilated over and above the supply needed by the tissues to compensate their natural waste circulates in the blood-current until excreted by the kidneys. But in order to be capable of transportation by the blood, it must first pass through the chemical laboratory of the body, the liver, and here be changed to compounds which can be excreted. All this work throws an immense burden upon the liver, and we cannot be surprised when it gets tired and lies down occasionally to rest. But when a larger amount of exercise is taken, all the tissues of the body have to supply the energy for this; they then greedily seize upon the food circulating in the blood-current to supply this waste, and thus relieve the liver of all that extra work placed upon it by the reverse conditions.

Theoretically, therefore, the relief for our patients should be brought about by diminishing and regulating the food-supply and increasing the exercise to supply the much-needed oxidation, and this is precisely what practical experience has proved to be the correct treatment. Quite a considerable number of persons seem to be poisoned directly by eating meat rich in blood and the organized nitrogenized compounds, such as creatinin, xanthine, and hypoxanthine; such meats are beef, veal, pork, and mutton, and it is reasonable to suppose that a considerable quantity of their extractives thrown into the blood may act as poisons. Patients thus affected usually suffer from persistent headache of an intractable character, and not amenable to the usual treatment, but they are promptly and permanently relieved when they abstain entirely from meat. Some of them can return to a moderate use of meat after withholding it for six months or a year, provided, however, they continue to take sufficient exercise. It may be laid down as a good general rule that all patients suffering from the diseases under consideration should eat little or no meat. They should eat largely of fruits and vegetables, and should endeavor to get as mixed a diet as is possible into each meal. They should also abstain entirely from wines and malt liquors; a little whiskey and water *with* the meal may be allowed. The wine-acids are especially objectionable, as they seem to interfere with prompt elimination, presumably because they abstract a considerable quantity of the alkalies of the fluids, so much needed in the transformation of the poisonous compounds into non-poisonous. The majority of these patients do not drink a sufficient quantity of water, and this habit should be inquired into and corrected. The habits of exercise should be carefully ascertained. It will be found that most all of these patients take little or none, and they must be made to take plenty of vigorous out-of-door exercise; bedroom gymnastics with dumb-bells and apparatus are not sufficient. Another great aid is found in maintaining the skin of the body in the greatest state of activity. No doubt the best method for this is cold bathing and brisk rubbing with coarse towels; this increases the circulation, removes the dead epidermis and stimulates the glands to greatest activity. In those who cannot endure the shock of cold water, a warm bath followed by a cooling douche, or Turkish and Russian baths, may be substituted. The object is to get the skin to do its utmost in aiding elimination, so as to relieve the kidneys of much of their work.

If these suggestions for the dietetic and hygienic management of the cases are faithfully followed and maintained, a cure will sooner or later be effected. But many patients claim that they have not the time to attend to all the necessary details, and want medicines for the relief of symptoms. Indeed, in almost all cases we have to give drugs for the relief of the most distressing symptoms, for a time at least, until the dietetic and hygienic changes have had a chance to alter the faulty chemistry. The object is to relieve the many distressing symptoms, which is best obtained by those drugs which will act as solvents and eliminants of the retained waste products. If we recall the chemistry as outlined at the beginning of this paper, we note that the acid urates chiefly, and to a lesser degree probably also creatin, xanthine, and hypoxanthine, together with some percentage of oxalic acid and its compounds, are the substances which we wish to get rid of. We also note that the compounds of the lighter metals, lithium, sodium, potassium, and ammonium, act as solvents of the uric acid series, and that hydrochloric and nitric acids act as solvents of the oxalic compounds. These facts established in the laboratory are proved to be correct when applied to the treatment of the cases under consideration. The writer has found from a considerable experience that *salicylate of lithium* is the most valuable drug at our command, and employs it usually in from ten- to thirty-grain doses given directly after meals. This drug differs from most of the other salts of salicylic acid in that it is well borne by most stomachs for a long time. Its good effects are very prompt, often relieving a severe headache and aches and pains in different parts of the body in a few hours, and after its use for a few days a general feeling of lightness and well-being is experienced. When the mineral acids are indicated I use a mixture of the strong nitromuriatic acid in glycerin and water, giving from three to five minims immediately before or after meals, preferably before meals. The relief experienced in many cases is often very prompt, and makes a deep impression on the patients. The *salicylate of soda* is not as good a drug as the salicylate of lithium in these cases, does not produce results as promptly, and is not well borne by most patients. The best mode of treatment is to give first a dose of calomel, preferably in divided small doses throughout the day, which is repeated once or twice a week according to the severity of the case. Another drug of great value is *salophen,* one of the coal-tar derivatives of more recent discovery. It has given instant relief in several cases of muscular rheumatism, rheumatic angina, and in some cases of severe urticaria. It is given in fifteen- to twenty-grain doses three or four times daily, is not disagreeable to the taste, and does not upset the stomach.

These comprise in a general way the drugs which the writer employs for these cases. The various local manifestations will of course often require special medications for the relief of more urgent symptoms, but, as a rule, if the above management is followed, most of the cases do well. Of course cases are met with which do not yield readily,—in fact, are very obstinate. In these I think that the conditions have lasted so long that organic changes have taken place and the normal functions of many organs are impaired, or they do not follow out faithfully the indicated treatment.

From the foregoing it is evident that the treatment of the diseases of the oral cavity, caused by the faulty elimination of tissue waste, must be constitutional and not local. Pyorrhœa alveolaris must of course be treated locally, but this can be only palliative, because we cannot reach the deep-seated pockets of suppuration under the gums. When the suppurative process has become well established, we can only give relief by syringing with peroxide of hydrogen and other antiseptics, but before the pus germs find a lodgement, and when we only have the spongy, bleeding gums, the condition should be curable by vigorous systemic treatment and strict attention to diet and hygiene. Even when a slight suppuration does exist, much can be done by combining the local treatment with the systemic, with a fair prospect for a cure in many cases.

If this faulty elimination goes on unchecked for many years, organic changes must be the inevitable result. The arterial system seems to be first affected. The fibrous tissue of the arteries is increased, phosphatic deposits take place, and a lessened elasticity of the vessels is the result. This in turn produces an obstructed circulation and consequent impaired nutrition of the various organs. From this parenchymatous changes occur, the parenchyma being replaced by fibrous tissue, and organic disease becomes established.

